# Role of junctional epithelium in maintaining dento-gingival adhesion and periodontal health

**DOI:** 10.3389/fdmed.2023.1144537

**Published:** 2023-03-08

**Authors:** Letícia Helena Theodoro, Valdir Gouveia Garcia, Edilson Ervolino, James Holcroft, Christopher A. McCulloch, Bernhard Ganss

**Affiliations:** ^1^ Department of Diagnosis and Surgery, School of Dentistry, São Paulo State University (UNESP), Araçatuba, Brazil; ^2^ Latin American Institute of Dental Research and Education (ILAPEO), Curitiba, Brazil; ^3^ Department of Basic Sciences, School of Dentistry, São Paulo State University (UNESP), Araçatuba, Brazil; ^4^ Faculty of Dentistry, University of Toronto, Toronto, ON, Canada

**Keywords:** periodontal tissues, junctional epithelium, periodontitis, enamel proteins, dento-gingival adhesion, review

## Abstract

The dento-gingival junction comprises multiple epithelia including the junctional epithelium (JE), which is the most coronally-located structural element of the dento-gingival junction that demarcates external from internal periodontal environments. After tooth eruption into the oral cavity, a specialized basal lamina is formed that provides a firm attachment of the JE to the enamel. This attachment prevents microbial species and oral debris from entering subjacent periodontal tissues. Here we discuss the expression of certain JE adhesion molecules and enamel proteins that maintain the health of the dento-gingival junction but that are perturbed in the pathogenesis of periodontitis. We also consider how evolutionary processes have influenced the development of the JE as a specialized adhesion that is well-suited for protection of the dento-gingival junction. A detailed understanding of the biology of the JE will deepen current models of dento-gingival adhesion, potentially clarify inter-patient variability of susceptibility to periodontitis and help to identify new roles of enamel proteins in periodontal regeneration.

## 1. Introduction

Teeth are one of the few tissues of mammals that penetrate protective epithelial layers. The sites at which teeth penetrate epithelia are potential entry points into deeper connective tissues for invasive and destructive microbial species, as seen in periodontitis and chronic systemic infections. Cognizant of the importance of the dento-gingival junction's sealing function around teeth for human health, we consider here the epithelia of the junction and how they have evolved to provide the first line of defense against infections of deeper periodontal tissues.

Gingival epithelia include oral epithelium (OE), sulcular epithelium (SE), and junctional epithelium (JE). Preservation of the attachment of the dento-gingival junction to teeth is particularly dependent on the integrity of the SE and the JE, which - unlike the OE - are non-keratinized. By means of its keratinized surface, the OE provides a physical barrier against the entry of microorganisms from the oral cavity. The SE plays an important role in immune surveillance at the gingival sulcus. The most coronal portion of the dento-gingival junction that attaches to the root surface is comprised of the JE, which separates external from internal environments and prevents the disruption and degradation of deeper periodontal tissues by debris and microbial species. The attachment of the JE to tooth surfaces is mediated by hemidesmosomes, the internal basal lamina and the cuticle. This structural complex provides a front-line defense against infections of the dento-gingival junction. In contrast to the OE and SE, the JE exhibits large intercellular spaces that may facilitate bacterial entry. This possible weak point in the overall integrity of the adhesive domains of the dento-gingival junction is countered by various innate defense mechanisms that include emigrating polymorphonuclear leukocytes, the rapid turnover of JE cells and the outward flow of gingival crevicular fluid that helps to clear microbial biofilms.

One of the high prevalence diseases prominently associated with JE dysfunction is periodontitis, an inflammatory disease driven by dysbiotic microbial biofilms that is a major cause of tooth loss because of progressive destruction of periodontal connective tissues (1). The initiation and rate of progression of periodontitis depends on the balance between bacterial species in biofilms, the immune status of the individual, environmental factors, oral hygiene habits, smoking, alcohol use, myriad genetic determinants and the functional and structural integrity of the JE. While the exact roles of the JE in the pathogenesis of periodontitis are not well-defined, it is known that marginal periodontitis lesions involve progressive and apically-directed detachment of the JE from the enamel and the conversion of the JE to a periodontal pocket epithelium. Below we describe the main structural characteristics of the JE including its expression of specific adhesion molecules and the role of the JE in maintaining the dento-gingival junction.

### 1.1. Structural characteristics of the JE

**Figure 1:**
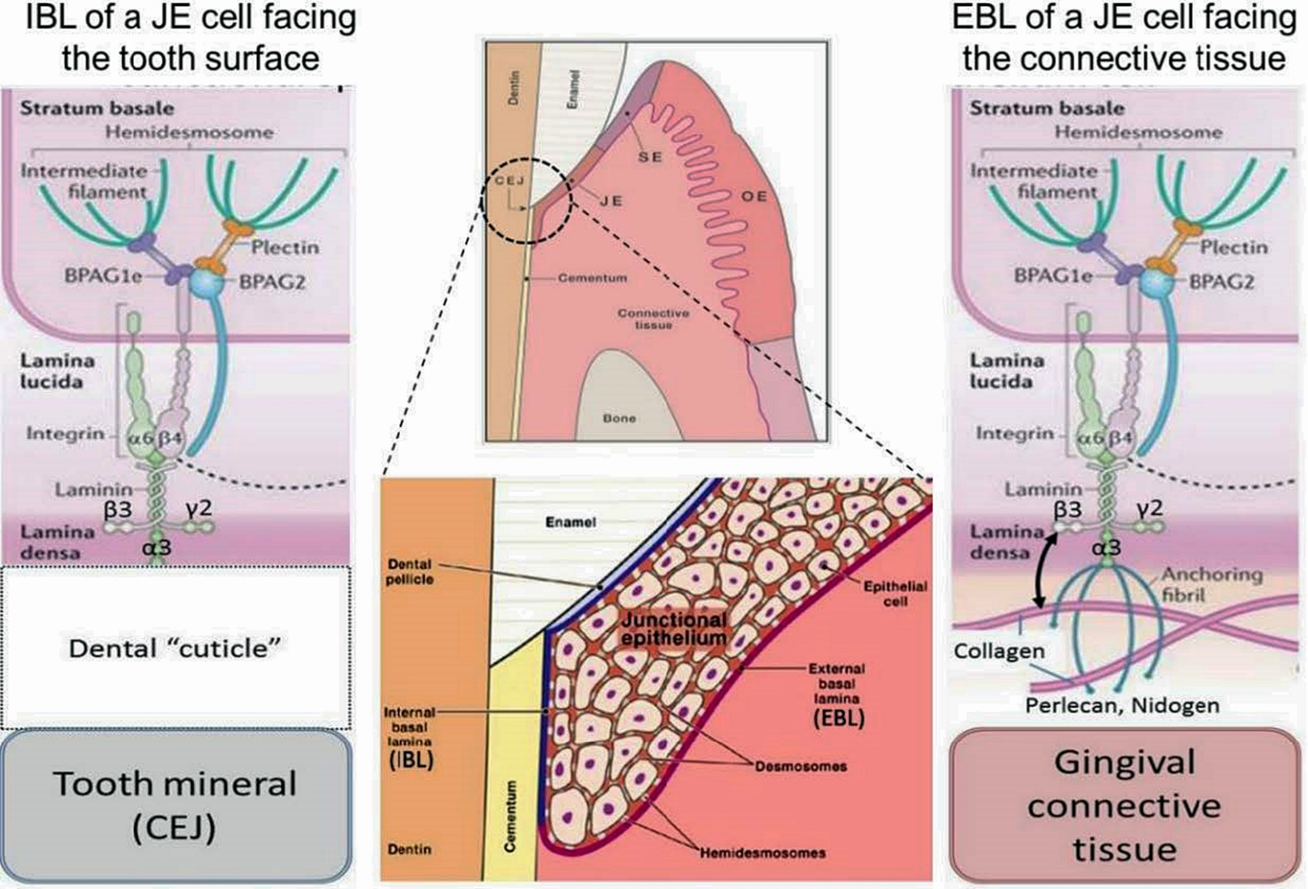
General morphological and molecular features of the junctional epithelium (JE). The JE is – like sulcular epithelium (SE) but unlike oral epithelium (OE) – non-keratinized. It has two distinct interfaces, one with gingival connective tissue (external basal lamina, EBL) and another with the mineralized tooth surface (internal basal lamina, IBL). Both interfaces employ hemidesmosomal adhesion complexes containing Plectin, BPAG1e and BPAG2 along with intermediate filaments. In the EBL, typical basal lamina components (e.g., Perlecan, Nidogen, Type IV Collagen) are present (right side of schematic), while in the IBL only LAM332 has been located. The nature and composition of the Dental “cuticle”, i.e the equivalent of the basal lamina at the IBL, has not been determined in much detail (left side of schematic).

The JE (Figure 1) is a specialized epithelium that mediates adhesion of the marginal gingiva to the surfaces of erupted teeth and dental implants (2). The JE originates from the odontogenic epithelium although it is not known whether those cellular elements that derive from the odontogenic epithelium are maintained in the JE over the organism's lifetime (3). The JE is classified as a stratified squamous epithelium and JE cell populations are uniformly arranged as layers aligned parallel to the root surface and exhibit wide intercellular spaces (4) While there are multiple layers of JE cells in the coronal portion of the attachment, the JE tapers apically (to 1 to 3 cell layers thick). There is no keratinizing epithelial cell layer towards the base of the gingival sulcus and in the interdental col. While the absence of keratinization may compromise innate defense mechanisms that protect against microorganisms and their metabolites, the rapid turnover of cells in the JE facilitates rapid repair and helps to maintain the integrity of the dento-gingival junction.

Two distinct basal laminae form at JE interfaces: the external basal lamina (EBL) connects JE cells to the underlying connective tissue while the internal basal lamina (IBL) attaches JE cells to the mineralized tooth surface, which in health is enamel. While some morphological aspects of the EBL and IBL are similar, their biochemical composition is quite different. The IBL is devoid of prominent components of typical basal laminae such as type IV collagen, perlecan and nidogen, for example (5). The structure and function of the EBL and IBL are influenced by the structure, physiological functions, chemical composition and mechanical properties of the tissue with which they contact. The detailed mechanisms that enable formation and regeneration of the JE are not well-defined (6) and because of the deficiencies of current cell culture systems for modeling JE and defining regulatory mechanisms, the molecular determinants of JE formation will likely provide considerable scope for investigation in the future.

### 1.2. Adhesion molecules

Integrins are heterodimeric cell adhesion receptors that mediate interactions between cells and extracellular matrix ligands, such as fibronectin and collagens. Integrins mediate inside-out and outside-in signaling from the extracellular matrix to the cell in a two-way process that regulates gene expression, cell proliferation, and cell migration. Integrins localize to the plasma membranes of JE cells where they interact with the IBL and EBL. In the IBL of the JE the *α*6ß4 integrin, which is enriched in hemidesmosomes, binds to laminin 332 (LAM332) to form a molecular adhesion complex (7). While the expression repertoire of integrins in healthy JE differs somewhat from basal cells in the OE, the JE does express some integrins that are similar to those expressed by keratinocytes in the oral mucosa and skin during wound re-epithelialization. Alterations in those integrins might influence periodontal tissue integrity. Besides, *α*vß6 integrin in JE may control periodontal inflammation *via* TGFß1 activation (8). Integrins and matrix molecules should regulate re-epithelialization during wound healing (8).

In addition to LAM332, other proteins that are not commonly found in basement membranes (e.g., type VIII collagen, versican, tenascin C) are present at the JE-tooth interface. In addition to E-cadherins that mediate intercellular adhesion in the JE, certain molecules such as Lymphocyte function antigen-3 and intercellular adhesion molecule-1 (ICAM-1 or CD54) are cell adhesion molecules that mediate the adhesion of JE cells to mineralized tissue surface.

### 1.3. Enamel matrix proteins

Amelotin (AMTN), odontogenic ameloblast-associated (ODAM) and secretory calcium-binding phosphoprotein proline-glutamine rich 1 (SCPPPQ1), together with LAM332 are involved in the supramolecular organization of the IBL and likely contribute to its adhesive capacity (9). Several additional genes, all located within the secreted calcium-binding phosphoprotein (SCPP) gene cluster, may also be involved in the fusion of reduced enamel epithelium with oral epithelium during JE morphogenesis (10). AMTN is a secreted protein expressed by ameloblasts during the maturation stage of enamel formation and exhibits very limited sequence similarity to other enamel proteins (11–13). AMTN expression persists in JE, which is similar to ODAM (13). AMTN in JE is restricted to the cell/mineral interface while ODAM is localized in pericellular zones of the JE (10, 14). As AMTN localizes to the basal lamina, degradation of AMTM could weaken the attachment of JE to tooth surfaces, thereby potentially increasing local susceptibility to marginal periodontitis lesions (15). Indeed, exposure to trypsin-like proteases and incubation with periodontopathic bacteria indicate that all constituents of the basal lamina, except SCPPPQ1, are rapidly degraded (9). Proteolytic enzymes, often produced by periodontal pathogens, can degrade AMTN, ODAM and LAM332 as constituents of the adhesive IBL, suggesting that this degradation could contribute to dento-gingival attachment loss and progression of periodontal disease (9).

Further, the localization and expression patterns of AMTN, ODAM and follicular dendritic cell-secreted protein (FDC-SP) may be modified at inflamed sites and especially at the internal basal lamina. The expression of FDC-SP and AMTN were increased in the JE of *P. gingivalis*-infected mice at early stages of inflammation but were decreased at sites of severe inflammation (13) while ODAM expression was increased at early stages and advanced stages of inflammation. In contrast, the expression of AMTN, ODAM and FDC-SP in *A. actinomycetemcomitans-*infected mice was not changed compared with non-infected control mice (13). In the human gingival tissues, AMTN was detected at the surface of the sulcular epithelium and JE in the non-inflamed and inflamed gingiva, and the localization did not change in the process of inflammation. ODAM and FDC-SP were more widely detected at the sulcular epithelium and JE in the non-inflamed gingiva. In the inflamed gingiva, localization of ODAM and FDC-SP was spread into the gingival epithelium compared with AMTN (13). These results suggested that AMTN, ODAM and FDC-SP were changed during inflammatory process, indicating that these proteins might play an important role during the inflammation process in periodontitis (13).

In addition to these enamel proteins, odontogenesis-associated phosphoprotein (ODAPH) is a separate, recently discovered enamel matrix protein that is detectable at the maturation stage of amelogenesis and is also a component of the IBL in the JE (16). Currently the adhesive function of ODAPH in the adhesive function of the dento-gingival junction is not defined.

### 1.4. Molecular interactions in the internal basal lamina of the JE

**Figure 2:**
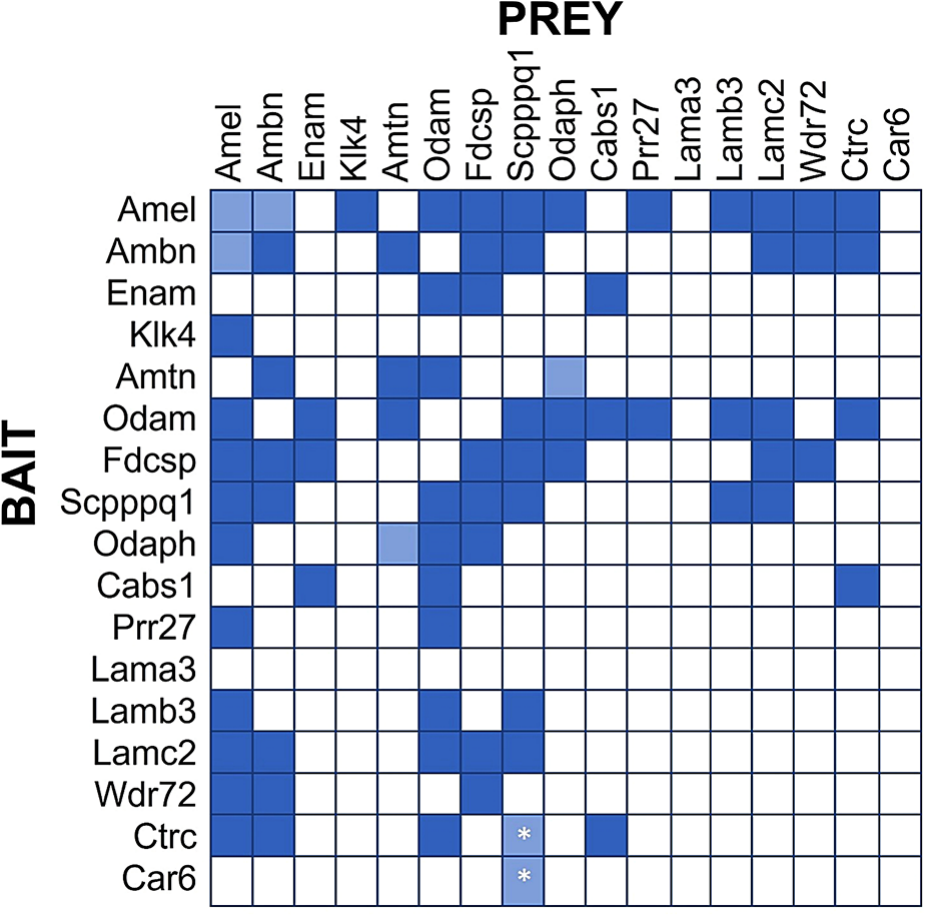
Protein Interaction Matrix determined by Yeast-two-Hybrid (Y2H) technology as described (16). Various constructs containing the listed proteins as Bait (left) or Prey (top) have been used. Dark blue squares indicate blue yeast growth on quadruple selection media, suggesting strong interaction. Light blue squares indicate blue yeast growth on triple selection media, suggesting weak interactions. All interactions were observed in both Bait-Prey and Prey-Bait combinations, except the interactions of Scpppq1 with Ctrc and Car6, which was only observed when Ctrc and Car6 were used as Bait and Scpppq1 as Prey, but not when Scpppq1 was used as bait and ctrc/Car6 as prey. These interactions require further confirmation.

Previous studies indicated interactions between laminins and enamel matrix proteins (17), which motivated our conduct of larger-scale protein-protein interaction screens using the yeast-two-hybrid system (Figure 2). Collectively, 17 proteins that are known or suspected to be involved in enamel formation and/or maturation were examined in this screen. These proteins include the well-known enamel proteins Amelogenin (AMEL), Ameloblastin (AMBN), Enamelin (ENAM), Kallikrein 4 (KLK4), along with the more recently identified proteins AMTN, ODAM, FDC-SP, SCPPPQ1 and ODAPH, calcium- and pH-regulating proteins CTRC and CAR6, LAM332 subunits, as well as proteins that are coded within the “enamel gene cluster” (Wdr72, CABS1, PRR27). Mutations in these genes are associated with developmental defects of enamel (18, 19), but no particular function, especially within the context of dento-gingival attachment, has been assigned to any of these proteins.

Our Yeast-2-hybrid protein interaction screen suggests several salient points (Figure 2):

1. Some proteins tend to interact with multiple other proteins (e.g., AMEL, ODAM, FDC-SP and SCPP), while other proteins do not appear to interact with many (or any) other proteins in this panel (e.g., LAMA3, Car6, KLK4).
2. The LAM332 subunits beta and gamma (Lamb3, Lamc2) only interact with a select number of enamel proteins, notably ODAM, AMEL, SCPPPQ1, and AMBN and FDCSP only interact with the Lamb3 subunit. The Lama3 subunit does not appear to interact with any enamel proteins, presumably because it engages primarily with the *α*6ß4 integrin, thereby possibly providing a connection with the hemidesmosome.
3. The interactions between enamel proteins found in the IBL are complex and likely involve many proteins (and possibly their proteolytic products) to form a three-dimensional network that specifically and dynamically mediates attachment of the gingiva to teeth.

Based on the results from this screen we tested various combinations of Laminin subunits and enamel proteins for their ability to mediate epithelial cell attachment to several solid surfaces. We found that only the combination of SCPPPQ1 and LAM332 can robustly increase adhesion strength and accelerate cell attachment to tissue culture plastic, hydroxyapatite and titanium surfaces (20). This is the first (and currently only) functional study of the molecules present in the IBL but will likely lead to more detailed investigations and hopefully a more detailed understanding of the molecular mechanisms of dento-gingival attachment at the IBL interface and possibly interfaces with dental implants. Indeed, the discovery of SCPPPQ1/LAM332-mediated cell attachment is a promising first step toward the design of new materials with improved attachment properties and toward solving the “percutaneous device dilemma” (21). Conceivably, this enhanced attachment is the result of increased density of hemidesmosomal attachment complexes (22) but further details are needed. Whether the previously reported SoxF-regulated formation of hemidesmosomal plaques (23) is affected by SCPPPQ1/LAM332 is not known.

### 1.5. Evolutionary aspects and lessons from animal models

Earlier phylogenetic comparisons that sought to clarify the evolutionary origin of the JE focused on structures that provide a “seal” against bacterial invasion of the dento-gingival junction. Analyses of the dentition in fish and reptiles and certain features observed in mammals have shown, perhaps not surprisingly, that the evolution of the dento-gingival junction and the JE is associated with specific physiological and mechanical needs (24, 25). The presence or absence of specific enamel-derived adhesion molecules (e.g., ODAM) is associated with specific structural features of the JE (26). Further, and as described at the outset of this review, it appears that the JE in part is derived from odontogenic (likely reduced enamel) epithelium and that the JE remains intact for a relatively long time after formation (7, 27).

Several animal models have been used to study the structural features of the JE. Rodents have been extensively used because of their rapid reproduction rate, economic husbandry, and the opportunity for precise genetic manipulations. Since incisors in rodents show the distinct feature of continuous eruption with enamel forming only on the labial side, the study of the JE in mice has focused largely on molar teeth. These studies demonstrate distinct features of the JE since the expression pattern of adhesion molecules in JE differs from OE and SE (28). As surgical procedures are difficult to perform in mice because of their small size, larger animal models have been used to investigate the cellular and molecular dynamics of the JE. Studies in dogs (29, 30), pigs (31) and non-human primates (32) have been particularly informative. Collectively, this research has revealed the intricate histological and immunological characteristics of the JE (6) and has underlined the importance of the developmental origins of the JE from multiple stem cell populations (33), which also operates during JE regeneration (34).

The evolution of the JE is likely constrained by two main determinants:

1. There is an important need to maintain a firm attachment at the most coronal part of the dento-gingival junction to isolate and protect underlying periodontal structures.
2. There is a need for effective immune surveillance in the gingival sulcus, which is facilitated by the permeable junctions between SE cells.

Animal models show that cell populations in the JE undergo rapid turnover, which enables distinction between JE, SE and OE and has helped to identify discrete progenitor cell populations involved in the maintenance of JE structure. Taken together, research from the past several decades indicates three distinct functions for oral epithelia that are associated with distinct structural characteristics: a) the OE provides an impermeable keratinized epithelial barrier, b) the SE enables enhanced immune surveillance, and c) the JE anchors the gingival tissue to the tooth, thereby isolating and protecting the underlying periodontal attachment apparatus.

From a molecular perspective and as described above, discrete protein repertoires are produced by cells of the reduced enamel epithelium and are located in the IBL between JE cells and the tooth surface. The next challenge will be to define the hetero-multimeric interactions between these proteins, including LAM332, integrins and components of the hemidesomosomal attachment apparatus. Recent data indicate that a complex consisting of SCPPPQ1 and LAM332 can accelerate and strengthen the adhesion of junctional epithelial cells to a variety of biologically relevant materials (20). Future progress will likely be facilitated by using primary JE cell cultures obtained from human tissues.

## 2. Conclusions

The JE is a complex, multifunctional structure that seals the dento-gingival junction around the necks of the teeth and prevents uncontrolled invasion of oral microorganisms. The complex structure of the adhesion complex of the JE to the tooth indicates its unique role in providing attachment of epithelial tissue to acellular mineralized tissue (such as enamel) and likely other materials that are widely used in transepithelial oral implants (e.g., titanium). The complex composition of the IBL as a specialized biological interface is currently being unraveled. The recently described ability of a Lam332/SCPPPQ1 complex to accelerate and strengthen adhesion of epithelial cells to solid surfaces may only be part of the overall adhesion mechanism. The ability to promote hydroxyapatite mineralization that has been documented for other proteins in the IBL such as AMTN (35) and ODAM (36) may play another important role in “anchoring” the multiprotein complex to the mineralized tooth surface. The specific roles of IBL proteins, individual or as part of multiprotein complexes, is an exciting new avenue to understand the mechanisms of dento-gingival attachment and to develop novel preventive and therapeutic strategies for periodontal diseases. As expression of enamel matrix proteins at the JE is likely altered at inflamed sites, these proteins may provide resistance to inflammation-associated degradation of the dento-gingival junction. Future research may focus on defining the cell adhesion molecules expressed in JE and whether their degradation contributes to susceptibility to periodontitis. Identifying the role of enamel proteins in regenerating of JE could provide new approaches for periodontal regenerative treatments.

## References

[1] Papapanou PN Sanz M Buduneli N Dietrich T Feres M Fine DH Periodontitis: consensus report of workgroup 2 of the 2017 world workshop on the classification of periodontal and peri-implant diseases and conditions. J Clin Periodontol. (2018) 45:S162–70. 10.1111/jcpe.12946 29926490

[2] Berglundh T Lindhe J Ericsson I Marinello CP Liljenberg B Thomsen P. The soft tissue barrier at implants and teeth. Clin Oral Implants Res. (1991) 2:81–90. 10.1034/j.1600-0501.1991.020206.x 1809403

[3] Yajima-Himuro S Oshima M Yamamoto G Ogawa M Furuya M Tanaka J The junctional epithelium originates from the odontogenic epithelium of an erupted tooth. Sci Rep. (2014) 4:4867. 10.1038/srep04867 24785116 PMC4007090

[4] Jiang Q Yu Y Ruan H Luo Y Guo X. Morphological and functional characteristics of human gingival junctional epithelium. BMC Oral Health. (2014) 14:30. 10.1186/1472-6831-14-30 24708739 PMC4234347

[5] Pöllänen MT Salonen JI Uitto VJ. Structure and function of the tooth-epithelial interface in health and disease. Periodontol 2000. (2003) 31:12–31. 10.1034/j.1600-0757.2003.03102.x 12656993

[6] Nakamura M. Histological and immunological characteristics of the junctional epithelium. Jpn Dent Sci Rev. (2018) 54:59–65. 10.1016/j.jdsr.2017.11.004 29755616 PMC5944073

[7] Shimono M Ishikawa T Enokiya Y Muramatsu T Matsuzaka K Inoue T Biological characteristics of the junctional epithelium. J Electron Microsc. (2003) 52:627–39. 10.1093/jmicro/52.6.627 14756251

[8] Larjava H Koivisto L Häkkinen L Heino J. Epithelial integrins with special reference to oral epithelia. J Dent Res. (2011) 90:1367–76. 10.1177/0022034511402207 21441220 PMC3215754

[9] Fouillen A Grenier D Barbeau J Baron C Moffatt P Nanci A. Selective bacterial degradation of the extracellular matrix attaching the gingiva to the tooth. Eur J Oral Sci. (2019) 127:313–22. 10.1111/eos.12623 31230388 PMC6771947

[10] Ganss B Abbarin N. Maturation and beyond: proteins in the developmental continuum from enamel epithelium to junctional epithelium. Front Physiol. (2014) 5:371. 10.3389/fphys.2014.00371 25309457 PMC4174742

[11] Iwasaki K Bajenova E Somogyi-Ganss E Miller M Nguyen V Nourkeyhani H Amelotin-a novel secreted, ameloblast-specific protein. J Dent Res. (2005) 84:1127–32. 10.1177/154405910508401207 16304441

[12] Moffatt P Smith CE St-Arnaud R Simmons D Wright JT Nanci A. Cloning of rat amelotin and localization of the protein to the basal lamina of maturation stage ameloblasts and junctional epithelium. Biochem J. (2006) 399:37–46. 10.1042/BJ20060662 16787391 PMC1570169

[13] Nakayama Y Kobayashi R Matsui S Matsumura H Iwai Y Noda K Localization and expression pattern of amelotin, odontogenic ameloblast-associated protein and follicular dendritic cell-secreted protein in the junctional epithelium of inflamed gingiva. Odontology. (2017) 105:329–37. 10.1007/s10266-016-0277-y 27807653

[14] Nishio C Wazen R Kuroda S Moffatt P Nanci A. Expression pattern of odontogenic ameloblast-associated and amelotin during formation and regeneration of the junctional epithelium. Eur Cell Mater. (2010) 10(20):393–402. 10.22203/eCM.v020a32 21154245

[15] Bartlett JD Simmer JP. New perspectives on amelotin and amelogenesis. J Dent Res. (2015) 94:642–4. 10.1177/0022034515572442 25900605 PMC4502783

[16] Li C Gao Y Xu Z Tian Y Mu H Yu C Expression and localization of amelotin, laminin *γ*2 and odontogenesis-associated phosphoprotein (ODAPH) on the basal lamina and junctional epithelium. J Mol Histol. (2022) 53:111–8. 10.1007/s10735-021-10026-w 34709488

[17] Holcroft J Ganss B. Identification of amelotin- and ODAM-interacting enamel matrix proteins using the yeast two-hybrid system. Eur J Oral Sci. (2011) 1:301–6. 10.1111/j.1600-0722.2011.00870.x 22243260

[18] El-Sayed W Shore RC Parry DA Inglehearn CF Mighell AJ. Hypomaturation amelogenesis imperfecta due to WDR72 mutations: a novel mutation and ultrastructural analyses of deciduous teeth. Cells Tissues Organs. (2011) 194:60–6. 10.1159/000322036 21196691 PMC3128158

[19] Smith CEL Poulter JA Antanaviciute A Kirkham J Brookes SJ Inglehearn CF Amelogenesis Imperfecta; genes, proteins, and pathways. Front Physiol. (2017) 8:435. 10.3389/fphys.2017.00435 28694781 PMC5483479

[20] Nouri S Holcroft J Caruso LL Vuong TV Simmons CA Master ER An SCPPPQ1/LAM332 protein complex enhances the adhesion and migration of oral epithelial cells: implications for dentogingival regeneration. Acta Biomater. (2022) 147:209–20. 10.1016/j.actbio.2022.05.035 35643199

[21] Fischer NG Aparicio C. Junctional epithelium and hemidesmosomes: tape and rivets for solving the “percutaneous device dilemma” in dental and other permanent implants. Bioact Mater. (2022) 18:178–98. 10.1016/j.bioactmat.2022.03.019 35387164 PMC8961425

[22] Park S. Exploring the molecular mechanism of dentogingival attachment mediated by laminin-332 and the enamel protein SCPPPQ1. Thesis, University of Toronto (2022). https://hdl.handle.net/1807/125382

[23] Oommen S Francois M Kawasaki M Murrell M Kawasaki K Porntaveetus T Cytoplasmic plaque formation in hemidesmosome development is dependent on SoxF transcription factor function. PLoS One. (2012) 7:e43857. 10.1371/journal.pone.0043857 22962592 PMC3433475

[24] Gaengler P Metzler E. The periodontal differentiation in the phylogeny of teeth–an overview. J Periodontal Res. (1992) 27:214–25. 10.1111/j.1600-0765.1992.tb01671.x 1608035

[25] McIntosh JE Anderton X Flores-De-Jacoby L Carlson DS Shuler CF Diekwisch TG. Caiman periodontium as an intermediate between basal vertebrate ankylosis-type attachment and mammalian “true” periodontium. Microsc Res Tech. (2002) 59(5):449–59. 10.1002/jemt.10222 12430171

[26] Springer MS Emerling CA Gatesy J Randall J Collin MA Hecker N Odontogenic ameloblast-associated (ODAM) is inactivated in toothless/enamelless placental mammals and toothed whales. BMC Evol Biol. (2019) 19:31. 10.1186/s12862-019-1359-6 30674270 PMC6343362

[27] Soda M Saito K Ida-Yonemochi H Nakakura-Ohshima K Kenmotsu S Ohshima H. Reduced enamel epithelium-derived cell niche in the junctional epithelium is maintained for a long time in mice. J Periodontol. (2020) 91:819–27. 10.1002/JPER.19-0269 31495928

[28] Hatakeyama S Yaegashi T Oikawa Y Fujiwara H Mikami T Takeda Y Expression pattern of adhesion molecules in junctional epithelium differs from that in other gingival epithelia. J Periodontal Res. (2006) 41:322–8. 10.1111/j.1600-0765.2006.00875.x 16827727

[29] Matsson L Theilade J Attström R. Electron microscopic study of junctional and oral gingival epithelia in the juvenile and adult beagle dog. J Clin Periodontol. (1979) 6:425–36. 10.1111/j.1600-051X.1979.tb01941.x 295290

[30] Kindlová M Trnková H. The vascular arrangement beneath the sulcular and junctional epithelium in different degrees of cellular infiltration of dog gingiva. J Periodontal Res. (1972) 7(4):323–7. 10.1111/j.1600-0765.1972.tb01721.x 4129099

[31] Marks SC Jr McKee MD Zalzal S Nanci A. The epithelial attachment and the dental junctional epithelium: ultrastructural features in porcine molars. Anat Rec. (1994) 238:1–14. 10.1002/ar.1092380102 8116883

[32] Braga AM Squier CA. Ultrastructure of regenerating junctional epithelium in the monkey. J Periodontol. (1980) 51:386–92. 10.1902/jop.1980.51.7.386 6771383

[33] Yuan X Chen J Grauer JA Xu Q Van Brunt LA Helms JA. The junctional epithelium is maintained by a stem cell population. J Dent Res. (2021) 100:209–16. 10.1177/0022034520960125 32985318 PMC8173348

[34] Tanaka K Tanaka J Aizawa R Kato-Tanaka M Ueno H Mishima K Structure of junctional epithelium is maintained by cell populations supplied from multiple stem cells. Sci Rep. (2021) 11:18860. 10.1038/s41598-021-98398-7 34552180 PMC8458500

[35] Abbarin N San Miguel S Holcroft J Iwasaki K Ganss B. The enamel protein amelotin is a promoter of hydroxyapatite mineralization. J Bone Miner Res. (2015) 30:775–85. 10.1002/jbmr.2411 25407797

[36] Ikeda Y Neshatian M Holcroft J Ganss B. The enamel protein ODAM promotes mineralization in a collagen matrix. Connect Tissue Res. (2018) 59:62–6. 10.1080/03008207.2017.1408603 29745811

